# Catarrh

**Published:** 1872-03

**Authors:** 


### CATARRH,

According to advertisements in the newspapers, is a dreadful disease, and certain to kill every man, woman, and child who has it, unless application is made at the places designated, and where a " perfect cure " will be " guaranteed " to every one who is willing to pay all the way from five dollars to five hundred, according to his greed or greenness. If a man is able to pay five hundred dollars, but will pay only fifty, why then he is taken in and done for to the extent of that fifty. If he is as green as grass, the proverb is illustrated, " A fool and his money are soon parted." The best "cure" in the world for catarrh is accomplished by doing two things — only two; it will cure three cases out of four, infallibly.

First, Let your nose alone.
Second, Avoid taking cold.

Catarrh is a cold, settling in the head or nose; and as long as it remains there, preserves the lungs from danger. If you snuffle anything whatever up the nose, in proportion as it arrests or diminishes the running from the nose, or eyes, or throat, in such proportion is it certain to show itself in the lungs, within a very few days, and even in a very few hours, sometimes.

This is the ordinary catarrh, which may last for days, or weeks, or months, and about which so many certificates of "cure" are flouted before the eyes of the public, in advertisements a mile long, in letters as tall as a court-house steeple, and as broad as a barn door.

One of the multitudinous dodges of these adventurous advertisers is to cause a violent diarrhœa; within a day or two the running at the nose and eyes ceases, because a drain has been set up in another direction. The patient gratefully and gladly writes a certificate of "cure," honestly believing it; but only to be "cured" until the next cold is taken, and all the old symptoms return. Keeping warm, avoiding colds, the bowels free, will cure all ordinary catarrhs. There is one kind rarely met with, comparatively speaking; but it is known by the matters discharged from the nose having a very offensive smell, indicating a scrofulous constitution; but as long as the nose runs freely the lungs are safe; stop the nose running, and the cough comes on, the forerunner of consumptive disease. A good physician will give a prescription to remove the odor only; he allows the discharge to continue, as a matter of safety, or sets up a steady drain from the body in another direction. If, then, the reader has catarrh, let it alone by all means, in the sense just stated.

